# Past, Present, and Future of Regulatory T Cell Therapy in Transplantation and Autoimmunity

**DOI:** 10.3389/fimmu.2019.00043

**Published:** 2019-01-31

**Authors:** Marco Romano, Giorgia Fanelli, Caraugh Jane Albany, Giulio Giganti, Giovanna Lombardi

**Affiliations:** ^1^Immunoregulation Laboratory, MRC Centre for Transplantation, School of Immunology & Microbial Sciences, King's College London, London, United Kingdom; ^2^Scuola di Specializzazione in Medicina Interna, Universita' degli Studi di Milano, Milan, Italy

**Keywords:** Tregs (regulatory T cells), transplantation, autoimmunity, cell therapy, clinical trial

## Abstract

Regulatory T cells (Tregs) are important for the induction and maintenance of peripheral tolerance therefore, they are key in preventing excessive immune responses and autoimmunity. In the last decades, several reports have been focussed on understanding the biology of Tregs and their mechanisms of action. Preclinical studies have demonstrated the ability of Tregs to delay/prevent graft rejection and to control autoimmune responses following adoptive transfer *in vivo*. Due to these promising results, Tregs have been extensively studied as a potential new tool for the prevention of graft rejection and/or the treatment of autoimmune diseases. Currently, solid organ transplantation remains the treatment of choice for end-stage organ failure. However, chronic rejection and the ensuing side effects of immunosuppressants represent the main limiting factors for organ acceptance and patient survival. Autoimmune disorders are chronic diseases caused by the breakdown of tolerance against self-antigens. This is triggered either by a numerical or functional Treg defect, or by the resistance of effector T cells to suppression. In this scenario, patients receiving high doses of immunosuppressant are left susceptible to life-threatening opportunistic infections and have increased risk of malignancies. In the last 10 years, a few phase I clinical trials aiming to investigate safety and feasibility of Treg-based therapy have been completed and published, whilst an increasing numbers of trials are still ongoing. The first results showed safety and feasibility of Treg therapy and phase II clinical trials are already enrolling. In this review, we describe our understanding of Tregs focussing primarily on their ontogenesis, mechanisms of action and methods used in the clinic for isolation and expansion. Furthermore, we will describe the ongoing studies and the results from the first clinical trials with Tregs in the setting of solid organ transplantation and autoimmune disorders. Finally, we will discuss strategies to further improve the success of Treg therapy.

## Introduction

Since the discovery in 1969 of the suppressor T cells (1), the regulatory T cells (Tregs) research field has undergone an incredible boom over the years. Studies on the biology of Tregs have attracted a lot of attention and our knowledge about their development and differentiation has increased enormously. The breakthrough in this field dates back to the discovery, in 1995, of a subset of thymus derived CD4^+^ T cells expressing high levels of IL-2Rα (CD25) able to protect thymectomized mice from autoimmunity (2). Since then, well-cited papers have shown the crucial role of Tregs in maintaining immune homeostasis (3) and preventing autoimmunity (4). Despite the improvement in Treg biology, nowadays there are no specific markers to characterize human Tregs. Differently from the mouse counterpart, the sole expression of CD25 and the transcriptional regulator forkhead box P3 (FOXP3) (5) is not sufficient for characterizing human Tregs, since effector T cells can upregulate these markers after activation. However, the methylation status of the Treg-specific demethylated region (TSDR) (6), a conserved non-coding element within the *FoxP3* gene locus, can be used for the identification of the “real” human Tregs (7). The analysis of TSDR methylation does not represent a suitable tool for their isolation; currently the expression of CD4, CD25, and lack of the α-chain of IL-7R (CD127) (8) are used for Tregs purification. In preclinical studies, human CD4^+^CD25^+^CD127^−^ Tregs have been shown to be effective in preventing Graft vs. Host Disease (GvHD) (9, 10), autoimmune diseases (11, 12) and delaying graft rejection (13, 14). The positive outcomes gave the rationale to apply Tregs for the treatment of human diseases and results from the first clinical trials with adoptively transferred Tregs were published in 2009 (15).

Solid organ transplantation represents the only treatment for end-stage organ diseases. Over the years, several strategies have been applied in order to improve transplantation outcomes and short-term graft survival (16). A better selection of donors and recipients associated with improved immunosuppressive schemes and patients' management has been crucial for ameliorating the graft survival in early stages. Long-term organ acceptance is a different story, remaining constant over the past decades (17). The immunosuppressive regimen, consisting of a combination of different drugs, aims to dampen the response of the immune system to the graft. Although successful in controlling the immune response early post-transplant, it is linked with detrimental side effects. Cardiovascular diseases, cancer, kidney failure and infections represent the main side effects that can cause graft loss and death (18). Long-term outcomes and finally operational tolerance are key for a successful organ transplantation. Different strategies are under investigation with the aim to reduce the use of immunosuppressive drugs. In this scenario, Tregs might represent a valid solution for controlling the immune response and inducing transplantation tolerance.

Autoimmune disorders are chronic diseases caused by the breakdown of tolerance against self-antigens. Usually they involve a specific region of the body such as the joints in rheumatoid arthritis (RA) or the pancreatic cells in type 1 diabetes mellitus (T1D). In other autoimmune diseases such as systemic lupus erythematosus (SLE) multiple areas are affected. The origin of autoimmune diseases is still a matter of debate; one hypothesis involves a failure in central and peripheral tolerance with the latter being associated with reduced Treg number or failure in their function (19). Furthermore, the combination of genetic and environmental risk factors has been implicated in the ontogenesis of autoimmunity as well (20). Similar to transplantation, immunosuppressive regimens aim to inhibit the activation of the immune system and reduce chronic inflammation. Different monoclonal antibodies targeting co-stimulatory molecules (21), cytokines (22), and lineage specific molecules (23) have been tested however, they all aim to target the immune and autoimmune responses leaving patients immunocompromised. For this reason, Tregs have been suggested as an effective tool for the treatment of autoimmune diseases.

## Tregs Ontogenesis

The summation of the research over the past years has demonstrated that the thymus is the crucial organ for the generation of Tregs (24). Animal models have shown that the differentiation of thymus-derived Tregs (tTregs) depends on T cell receptor (TCR) signaling, particularly the strength and duration of the signal (25). Despite technical limitations, this has been confirmed in humans as well (24). In thymus, immature CD4 single positive (SP) cells receive a TCR signal of varied strength, which will drive their fate. Following a TCR signal of high strength, most CD4 SP cells undergo negative selection, whereas those receiving TCR signals of intermediate strength are able to escape deletion and are committed to differentiate into Tregs (26). Nevertheless, whether there are differences between TCR signals for conventional T cells (Tconv) and Tregs is still an open question. Some pieces of evidence so far support the idea of “quantitative” difference in signaling, but it is also plausible that TCR signals might be “qualitatively” different. Beyond TCR signaling, CD28 is also crucial in the generation of tTregs. In fact, both CD28–deficient and CD80-CD86-deficient mice have decreased number of Tregs (27). Several other factors, including NFAT/AP1, ICOS/ICOSL and thymic stromal lymphopoietin (TSLP) are involved in the transcriptional control of human Treg differentiation (28–30). FOXP3 expression requires the presence of γ chain cytokines (IL-2, IL-15, and IL-7) and the reduction of PI3K-mTOR signaling pathway. Mice deficient in IL-2 or IL-2Rα have decreased number of FoxP3^+^ thymocytes, while ablation of IL-15 and IL-7 alone does not have such effect (5). The essential role of IL-2 in the generation of Tregs has been confirmed in humans as well (29). Phosphatidylinositol 3-kinase (PI3K) is induced by TCR and CD28 signaling and through the activation of Akt-mTOR pathway, antagonizes FOXP3 expression, thereby inhibiting the development and suppressive function of tTregs (31). Conversely, the hyper-activation of this pathway in Tconv cells has been suggested as a possible mechanism to overcome Treg suppression (32). In recent years, it has been demonstrated that the demethylation status of a TSDR is essential for human Treg lineage maintenance (6). Therefore, both FOXP3 expression and TSDR demethylation are essential for tTreg lineage commitment. Together, these findings demonstrate that TCR signaling in combination with other cell-intrinsic and extrinsic signals orchestrates human tTreg cell differentiation.

In addition to tTreg, naïve FOXP3^−^CD4^+^ T cells can differentiate in the periphery to become FOXP3^+^cells, which are known as induced Tregs (iTregs) or peripheral Tregs (pTregs). Differently from tTregs, the generation of iTregs is likely promoted by non-self-antigens (allergens, food, microbiota) (33). It has been also shown that a distinct TCR repertoire and ligand specificity support the generation of iTregs. These TCRs are of high affinity and their sequences only partially overlap with the TCRs used by tTregs. Additionally, an efficient induction of FOXP3 and iTreg generation occurs *in vivo* upon TCR stimulation together with suboptimal co-stimulation (decreased CD28 signaling) (34). Therefore, TCRs that recognize antigens to which an organism is chronically exposed promote the generation of iTregs. Low levels of costimulatory molecules in the presence of anti-inflammatory molecules secreted by tolerogenic DC cells (tDCs) promote the differentiation of iTreg cells as well (35). Beyond TCR signaling and suboptimal co-stimulation, the polarization of naïve CD4^+^ T cells into iTregs requires the combination of TGF-β and IL-2 (36). For example, several animal studies have shown that TGF-β/TCR-mediated iTreg cell generation is strictly dependent on IL-2 signaling. IL-2 promotes the activation of *Foxp3* locus through STAT5 and constrains the differentiation of activated CD4^+^ T cells into Th17 cells (37, 38). So far, *in vitro* TGF-β-induced iTreg cells have been considered a valid approach to study the development of iTreg cells *in vivo*. However, this experimental method fails in recapitulating the epigenetic and transcriptional characteristics of *in vivo* induced iTreg cells, namely transient suppressive ability and unstable FOXP3 expression, precluding therefore their use in the clinic (39). Inducible T regulatory type 1 (Tr1) cells are a subset of iTregs characterized by the ability to produce the immunosuppressive cytokine IL-10 (40). Tr1 can only transiently up-regulate FoxP3 following stimulation. These cells have been shown to maintain peripheral tolerance, modulate effector T cell responses in several autoimmune diseases and prevent allograft rejection (40). The possibility of generating *in vitro* expanded Ag-specific Tr1 cells has encouraged their clinical use in autoimmunity and Graft vs. Host Disease (GvHD).

Current evidence indicates that tTregs and iTregs are designated to play different roles in different tissues. Owing to the nature of iTreg differentiation induced by non-self- antigens and a particular TCR signaling combined with other signals, such as TGF-β and IL-2, these cells are assumed to be more functional for maintaining mucosal tolerance. iTregs may therefore control immune responses to commensal antigens and prevent allergic-type reactions.

## “Heterogeneity of Tregs”

Tregs in circulation are considered heterogeneous, this is mainly due to their plasticity and the capacity to acquire features specific to the type of immune response they control. In the literature, Tregs are divided in subpopulations according to the sites of differentiation and the expression of well-known functional markers. However, this does not allow a full distinction due to the overlap and redundancy between many of these parameters. For the first time in 2009, Miyara et al. demonstrated that human Tregs consists of three subpopulations based on their expression levels of FOXP3 and CD45RA (41). Tregs were classified as naive/resting (CD45RA^+^FoxP3^low^), effectors (CD45RA^−^FoxP3^high^), and cytokine-producing (CD45RA^−^FoxP3^low^). Naive Tregs are considered the “real Tregs” arising from thymus with a fully demethylated *FoxP3* locus. Effectors Tregs are the active population *in vivo* while the cytokine-producing Tregs include those cells able to produce pro-inflammatory cytokines like IL-17 and IFN-γ but still able to suppress. More recently, human Tregs named T helper-(Th-) like Tregs have been described (42). These memory Tregs mirror the classical CD4^+^ Th population expressing the same chemokine receptors CXCR3, CCR6 and CCR4, typically expressed by T-bet^+^-Th1, RORgt^+^-Th17, and GATA3^+^-Th2, respectively. We have fully characterized these subsets showing their cytokine production, suppressive and migratory ability (43). *In vitro*, all Th-like Tregs are functional with no preferential suppressive ability toward the cognate Th counterpart. This highlights the importance of Th/Tregs co-localization for the control of the immune system activation. The ontogenesis of these subsets is still under debate due to the high Treg plasticity, which can be detrimental in the setting of autoimmune diseases (44). In this scenario, Tregs acquire Th phenotypes associated with a reduced function despite maintaining Foxp3 expression and demethylation. The frequency of Th1-like Tregs is increased in patients with T1D (45), multiple sclerosis (46) and autoimmune hepatitis (47) and it is associated with a reduced suppressive ability. Similarly, Th2-like Tregs are increased in the skin but not in the peripheral blood of patients with systemic sclerosis (48). Whereas, Th17-like Tregs are increased in psoriasis (49) patients and inflammatory bowel diseases (50, 51) with no loss of function. However, the origin and the fate of Th17-like Tregs is matter of debate as some authors suggested that they might represent a transient stage in the differentiation of Tregs into Th17 cells (52). Under inflammatory conditions and autoimmune diseases, FOXP3^+^ Tregs can convert into Th17 thus impairing immune homeostasis and contributing to the progression and pathogenesis of the disease (44). As already mentioned, the demethylation of the TSDR region is the key determinant for Treg stability and function. FOXP3 is known to neutralize RORγt transcription, the master transcription factor of IL-17 producing Th17 cells (53). Therefore, a highly stable Foxp3 expression *in vitro* is associated with a small risk for IL-17 production *in vivo* under inflammatory conditions.

## Mechanisms by Which Tregs Suppress

**Figure 1:**
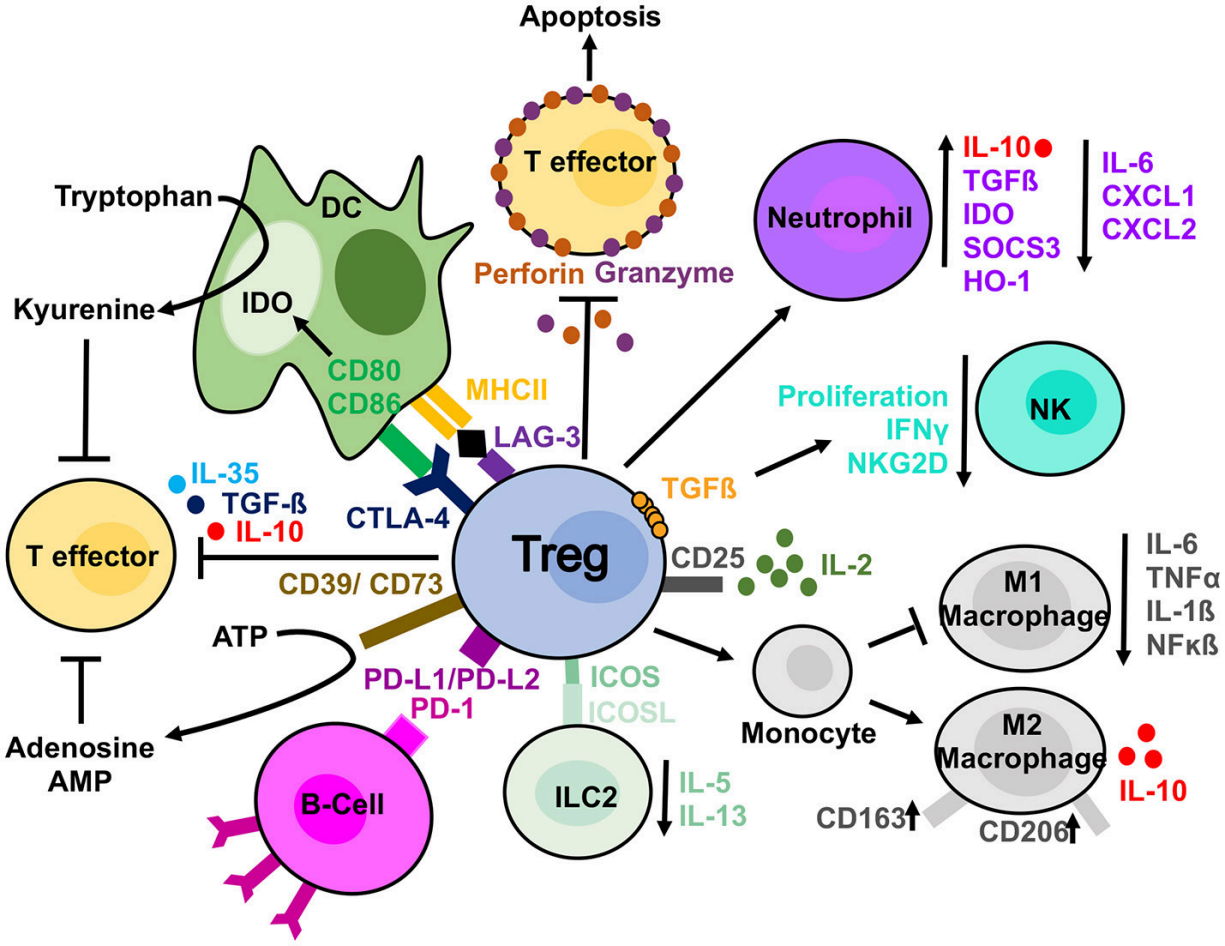
Treg suppressive mechanisms. Tregs are able to suppress different cell types by direct and indirect mechanisms. Tregs can produce anti-inflammatory cytokines (IL-10, IL-35, and TGFβ) affecting T cells. In addition, they release perforin and granzyme, which damage target cell membrane leading to apoptosis. They can sequester, by the high expression of CD25, IL-2 from the microenvironment reducing effector T cells proliferation. IL-2 starvation reduces NKs from proliferating and exhibiting effector functions as well. Furthermore, NKs can be directly affected by Tregs in a membrane bound TGF-ß dependent manner. Tregs have been observed to have a direct effect on B-cells via PDL1/PD-1 interaction and DCs via both CTLA-4 and LAG-3. CTLA-4 blocks co-stimulation reducing CD80/CD86 expression and it induces upregulation of IDO. The expression of CD39 on Tregs mediate the conversion to ATP to adenosine and AMP and reduce T effector proliferation. Tregs can also skew monocyte toward M2 macrophages and prevent their differentiation in pro-inflammatory M1 macrophages. They can similarly induce a suppressive phenotype in neutrophils and reduce ILC2 cytokine secretion.

The mechanisms used by Tregs to suppress different immune cells can either be considered direct whereby Tregs themselves elicit a direct response on a target cell, or indirect, in which a third-party cell or molecule is affected and in turn suppresses the target cell (54). Examples of direct mechanisms include the secretion of cytokines such as IL-10, TGFβ and IL-35 and the production of granzyme and perforin, enzymes leading to apoptosis in target cells (54). Indirect mechanisms include the expression of CD39/CD73, which deplete the microenvironment of extracellular ATP via the generation of adenosine and AMP, molecules with immunosuppressive effects (54). Alternatively, Tregs can influence changes in the microenvironment due to their high expression of CD25. The high expression of this receptor enables Tregs to uptake more IL-2 and “starve” the surrounding cells of this cytokine (55). When considering whether there is a “dominant” mechanism of suppression utilized by Tregs, it is important to understand that different mechanisms are utilized preferentially for the vast variety of target cells and microenvironments in which Tregs act. Below, we explore, more specifically the methods used on several “key” cell types from both the innate and adaptive arms of the immune system (Figure 1).

### T-Lymphocytes

This includes of CD4^+^ and CD8^+^ cells. Tregs suppress CD4 T cell activation and proliferation by contact-dependent and contact-independent mechanisms [extensively reviewed in (56)]. An important factor for Treg suppressive ability on other T cells is their localization. This is in line with the mutual distribution of the Th-like Tregs and classical Th cells observed by us in human thymus, spleen, liver, and colon (43). Additionally, Tregs can also influence proliferation, activation and apoptosis of CD8^+^ T cells (57, 58). As a result, the induction of high affinity effector and memory CD8^+^ T cells is reduced.

### B-Lymphocytes

B-Lymphocytes are important components of the adaptive immune system acting largely by the production of antibodies; however, they can act as APCs as well. B-cells require activation by T-cells following antigen recognition. Upon activation, B-cells differentiate into “effector” plasma cells which can produce antibodies. Tregs have the potential to suppress autoreactive B-cells in an antigen-specific manner and prevent the production of harmful autoantibodies. This suppression requires PD-1 expression on autoreactive B cells and expression of the two PD-1 ligands (PDL-1 and 2) on Treg (Figure 1) (59). In addition, Tregs are able to kill B-cell by releasing granzyme B and perforin (60).

### Dendritic Cells

Dendritic cells (DCs) play a critical role in the regulation of the adaptive immune response by activating resting naïve T cells. In the setting of transplantation, they are key in mediating graft rejection through direct, indirect and semi-direct presentation [extensively reviewed by us in (61)]. Similarly, DCs have been involved in the pathogenesis of many autoimmune diseases (62). Animal studies have shown that Tregs can interact with DCs in a leukocyte function-associated antigen-1 (LFA-1) dependent manner (63) and down-regulate the expression of CD80/CD86 on target cells by CTLA-4 (Figure 1). However, in CTLA-4 KO-mice Tregs could still suppress via compensatory mechanisms involving TGF-β and IL-10 (64). Furthermore, Tregs have the ability to increase the expression of Indoleamine 2,3-dioxygenase (IDO) in DCs (Figure 1) (56). This enzyme catalyzes the degradation of tryptophan to kyneurine leading to starvation of effector cells. Unlike effector T cells, Tregs can express LAG3 a homolog of the CD4 receptor. It binds to MHC-II with a significantly higher affinity than CD4 mediating the activation of PI3K/AKT, p42/44ERK, and p38MAPK pathways (65). As a result, DCs exhibit an increased expression of co-stimulatory molecules but reduced capacity to capture the antigens. In addition to these cell-contact depended mechanisms, Tregs can disrupt the microenvironment in the immunological synapse provided by DCs and essential for T cells proliferation. In detail, Tregs act either reducing the limiting enzyme for glutathione (GSH) synthesis or consuming extracellular cysteine (66, 67).

### Monocytes

Under inflammatory conditions, monocytes migrate into the tissue, where they differentiate into dendritic cells or tissue-resident macrophages. Monocytes constitute the major cellular component in inflamed tissues and their regulation might be key in reducing chronic inflammation. Tregs have been demonstrated to directly act on monocytes inhibiting their cytokine secretion, differentiation and antigen presenting function. Following co-culture with Tregs, monocytes exhibited classical features of M2 macrophages such as increased expression of CD206 (mannose scavenger receptor) and CD163 (hemoglobin scavenger receptor), simultaneously these cells showed a reduced capacity to respond to pro-inflammatory stimuli as demonstrated by decreased production of IL-6 and TNF-α and decreased NF-kB activation (68) (Figure 1). Recently we have shown that *ex vivo* expanded Tregs are more efficient in skewing monocytes toward a tolerogenic phenotype. Of note, monocytes co-cultured with expanded Tregs showed a reduced capacity to increase detrimental IL-17 producing T-cells when compared to freshly isolated Tregs (69). This mechanism was due to a decreased expression of CD86 by Tregs-conditioned monocytes.

### Granulocytes

Granulocytes are a group of cells belonging to the innate immunity. Among them, neutrophils are the first to respond to sites of inflammation where they phagocytose pathogens, release proteolytic enzymes, and produce antimicrobial peptides. Dysfunction of these cells results in sustained inflammation which can cause a number of pathological conditions including sepsis (70) and autoimmune diseases (71). Tregs directly affect neutrophils (Figure 1) limiting their accumulation. They do this by decreasing the expression of chemoattractants, CXCL1 and CXCL2, thus preventing aberrant skin infiltration (72). Furthermore, Tregs can also induce a more “immuno-suppressive” phenotype in neutrophils, thus skewing the microenvironment toward a less inflammatory one. Neutrophils co-cultured with Tregs produced more IL-10 and TGF-β together with a decreased IL-6 production (73). In addition, Tregs induced the expression of heme oxygenase-1, IDO and the suppressor of cytokine signaling 3 molecule (SOCS3) (73).

Basophils are another subgroup of granulocytes which are critical in mediating allergic and inflammatory responses. A recent publication showed that Tregs were able to activate resting basophils inducing their expression of CD69, CD203c, and CD13. Further to these, activated basophils were able to release IL-4, IL-8, and IL-13 (74).

### Innate Lymphoid Cells (ILCs)

Recently described, ILCs are a subset of the innate immunity arising from a lymphoid precursor. They are divided into three groups depending on the expression of specific transcription factors and cytokines [extensively reviewed in (75)] Natural Killer cells (NKs) release cytokines, recruit other immune cells via IFN-γ and TNF-α production and have direct cytotoxic activity. Interactions between NKs and Tregs have been reported (76). During pregnancy, Tregs suppress NKs to create a tolerant environment favoring the implant (77) while in tumors Tregs have the potential to block NKs generating an immune-suppressive environment, which favors cancer cell survival (76). Mechanisms of control exerted on NK cells by Tregs have been investigated (Figure 1). Following activation, Tregs suppress NKs via membrane bound TGF-β (78). This suppression results in the inhibition of NK cells' effector functions and a down regulation of NKG2D receptors on cell surface. Further to this, by restricting the availably of IL-2 in the microenvironment, Tregs prevent the NKs from proliferating, secreting IFN-γ and enhancing missing self-recognition (76). Tregs can also affect ILCs2. In particular, iTregs but not tTregs have the ability to suppress ILC2 function (Figure 1) preventing their secretion of both IL-5 and IL-13 in an ICOS/ICOSL dependent-manner (79).

## Tregs Isolation and Expansion

Tregs can be isolated in large scale from either peripheral blood (PB) (80), umbilical cord-blood (UCB) (81) or thymus (82). To date, only Tregs isolated from UCB and PB have been tested in the clinic. The isolation under GMP condition was carried out by “CliniMACS” system (CliniMACS TM Instruments, Miltenyi Biotec) a clinical-scale magnetic enrichment of cells in a closed and sterile system. The protocol consisted in either the depletion of CD8^+^ and CD19^+^ cells or CD8^+^ only followed by an enrichment of the CD25^+^ fraction. Although the CliniMACS has been used by several groups for isolating Tregs, cell purity represents an important limitation. As reported by Di Ianni et al., only 80% of the cells were FOXP3^+^ due to the presence of cell contaminants (83). The presence of non-FOXP3 cells might be deleterious especially if the generation of antigen specific cells has been planned. Another method used to isolate Tregs in the clinic is the flow cytometry-based purification. Similar to the cell sorter routinely used in the research field, cells can be isolated according to the expression of selected cell markers. To date, flow-sorted Tregs have been used in clinical trials outside the EU (84, 85) where cells have been selected according to the expression of CD4, CD25, and CD127 with high purity (>99%). However, in the last few years, different companies have started to develop GMP compatible cell sorter and fluorescent antibodies. This will allow the isolation of CD4^+^CD25^+^CD127^low^CD45RA^+^ Tregs, a subset more suitable for long-term expansion due to an epigenetically stable FOXP3 expression and an increased resistance to Th17 conversion (86). Moreover, this method does not allow the isolation of activated effector cells that usually express intermediated levels of CD25 and are increased in autoimmune diseases. However, due to the high number of processed PBMCs at the beginning, a pre-enrichment for CD4+ cells might be necessary. As a consequence, the cost for a single preparation will increase considerably.

Another challenge for the research groups aiming to start/develop new clinical trials with Tregs has been their low number. In PB, Tregs are 5–10% of all the circulating CD4^+^ T cells. Although in CB and thymus the number of Tregs is higher, the infusion of a large cell number has been difficult to achieve. The first clinical trial in GvHD used freshly isolated Tregs (87). However, during the same period our group and others developed a clinically scalable protocol for their expansions (88, 89). Tregs are now routinely expanded *ex-vivo* in 36 days using anti-CD3/CD28-coated beads in the presence of high dose of IL-2 (polyclonal expansion) (90). In detail, our protocol involves the use of rapamycin, an immunosuppressant that inhibits the mechanistic target of rapamycin (mTOR) protein kinase. Rapamycin added during the course of the culture, inhibits exclusively the proliferation of effector T-cells. In addition, by blocking the signaling through AKT–mTOR-SMAD3, rapamycin favors FOXP3 upregulation (91). Rapamycin confers to the expanded Tregs higher stability and suppressive capacity; of note, Tregs from TD1 patients and patients with cirrhosis on a waiting list for a transplant expanded in the presence of rapamycin recover their suppressive ability (80, 92). Together with rapamycin and IL-2, Leventhal's group has developed a protocol to expand Tregs in the presence of TGF-β (93). At the end of the expansion, their product was more suppressive compared to the use of rapamycin alone and TSDR more demethylated compared with the freshly isolated counterpart. All-trans retinoic acid (ATRA) is another molecule that can be used for Tregs expansion. Similarly to TGF-β, ATRA can induce the generation of Tregs (94). During Treg expansion this molecule is essential for the upregulation of chemokine receptors responsible for gut homing like CCR9 and integrin-α4β7 (89). Due to its peculiarity, ATRA will be used for the expansion of Tregs in one of our clinical trials, “TRIBUTE” aiming to evaluate the infusion of *ex-vivo* polyclonally expanded naïve Tregs in patients with Crohn's Disease. Umbilical cord blood-derived Tregs have been isolated and expanded for the treatment of GvHD (95). Cells were stimulated with anti-CD3 mAb and artificial APC consisting of K562 cell lines (KT) engineered to express CD86 and the high affinity Fc Receptor (CD64) (KT64/86). During the 19 days culture, cells were supplemented with 300 IU/mL of IL-2. Finally, the generation and expansion of alloantigen specific Treg is a promising strategy that can be tested soon in the EU with the advent of the GMP-cell sorter. Our preclinical protocol for the generation and expansion of antigen-specific Tregs involves the co-culture of Tregs with CD40-activated allogeneic B-cells or donor-derived DCs in the presence of IL-2. Antigen-specific Tregs have been shown to be more powerful in suppressing alloimmune responses *in vitro* and *in vivo* compared to the polyclonally expanded Tregs (96).

## Clinical Trials Enrolling Regulatory T Cells

After 10 years from the first Treg infusion in patients with GvHD, several phase I or phase I/II clinical trials have been completed or started. They aim to test the safety, feasibility and efficacy of Treg infusion in the setting of solid organ transplantation (97) GvHD (98) and autoimmunity (84). In the next sections, we describe results from published studies in the setting of autoimmune diseases and transplantation giving an overview of the main clinical trials that are ongoing.

### Tregs in Autoimmunity

The first-in-man clinical trial adopting Tregs in autoimmune diseases was the “CATS1” study, the results of which were published in 2012 by Desreumaux et al. (99) In this phase I/IIa, open-label, multicentric trial, 20 patients with active and symptomatic refractory Crohn's Disease were divided in 4 dose cohorts receiving a single infusion of 10^6^-10^9^ Tr1. Firstly, PBMCs were cultured in the presence of ovalbumin in medium supplemented with supernatant derived from Drosophila Schneider 2 (S2). S2 cells were previously transfected to produce IL-2 and IL-4 and express trans-membrane mouse anti-human CD3, CD80, and CD58. After 7 days of culture growing clones were harvested and tested for antigen specificity and Tr1 cell identity before being expanded on Drosophila feeder cells. Cell infusion has been considered safe and, the reported unexpected severe adverse events were correlated to the natural history of the disease rather than the treatment. In 2014 Marek-Trzonkowska et al., published the results from a prospective, non-randomized phase I trial with the purpose to evaluate safety and feasibility of the infusion of autologous *ex-vivo* expanded polyclonal Tregs in patients with recently diagnosed T1D (85). Twelve patients aged 7–18 were enrolled and compared with ten patients who met the eligibility criteria, whose blood could not be collected due to inappropriate venous access. Fresh blood (250 mL) was collected and Tregs (CD4^+^CD25^+^ CD127^low^) were sorted and expanded using antiCD3/CD28 coated-beads, IL-2 and autologous serum without using rapamycin. Three patients received a single dose of 10 × 10^6^ cells/kg, other three patients received 20 × 10^6^ cells/kg, while six patients were offered a double dose up to a total 30 × 10^6^ cells/kg. The last group was composed of those patients who showed good laboratory and metabolic response, but symptoms of disease progression after 6 months from the first infusion. After 1 year of follow-up, safety was proved by the absence of serious adverse events and eight patients showed signs of clinical remission, among those, two remained insulin-independent. Conversely, the untreated patients remained insulin-dependent with lower C-peptide levels.

In 2015, Bluestone et al. published results form an open-label, interventional phase I clinical trial conducted at University of California (San Francisco) and Yale University (84). They aimed to determine safety and feasibility of intravenous infusion of *ex-vivo* expanded autologous polyclonal Tregs in patients with T1D. Fourteen recently diagnosed T1D patients, six females and eight males, were divided into four dose cohorts, ranging from 0.05 × 10^8^ cells for cohort one to 26 × 10^8^ cells for cohort four, with 8-fold dose increase in each cohort. Two weeks before the planned single infusion, 400 ml of fresh peripheral blood was collected; Tregs were isolated via Cell Sorting and cultured for 14 days in the presence of antiCD3/CD28 coated-beads and IL-2. Out of the sixteen eligible patients, two did not receive treatment, due to failure in expanding Tregs. After a mean follow-up of 31 months, only three severe adverse events were reported (two hypoglycaemias, one ketoacidosis), while no opportunistic infections were observed. None of the total reported adverse events (mild to severe) was related to cell infusion. Seven patients (cohort 3 and 4) received deuterium-labeled Tregs in order to track the infused cells. After 1 day post infusion, Tregs could be detected in circulation with a peak observed by 7 to 14 days. The percentage of deuterium labeled Tregs dropped to 25% after 3 months remaining stable up to 9 months. After 1 year, deuterium was still detectable in four patients with no evidence of differentiation of Tregs into T effectors. Although this study showed a feasible and safe therapeutic approach to T1D, with stably suppressive Tregs, the small number of treated patients as well as the early phase of the trial could not help to shed light on the optimal dose and the impact of Tregs on the function of islet cells. To address these points, a multicentre phase II randomized, placebo-controlled double blind clinical trial **(NCT02691247)** is underway, with the purpose to evaluate, in young patients, safety and effect on beta cell function of a single dose (low 2.5 × 10^6^/kg vs. high 20 × 10^6^/kg compared to placebo) of autologous *ex-vivo* expanded polyclonal Tregs. Another ongoing phase I clinical trial **(NCT02772679)** is evaluating safety and optimal dosing of a single infusion of autologous *ex-vivo* expanded polyclonal Tregs (CD4^+^CD25^+^CD127^low^) followed by injection of IL-2 in patients with T1D. The enrolled patients will be allocated into two dose-cohorts receiving 3x10^6^ cells/kg and 20 × 10^6^ cells/kg, respectively. IL-2 (1 × 10^6^ IU) will be administered subcutaneously, daily, for the 5 consecutive days post infusion and repeated after 1 month. The primary outcome will be the occurrence of any adverse events and the evaluation of distribution of deuterium-labeled Tregs.

In 2018, Dell'Era et al. published results from **NCT02428309**, a phase I non-randomized, open-label study originally set to evaluate escalating single dose of autologous, FACS-sorted and *ex-vivo* polyclonally expanded Tregs (1 × 10^8^, 4 × 10^8^, and 16 × 10^8^ cells, respectively) in adult patients with active cutaneous Lupus (100). Due to screening failures and comorbidity burden, a single patient was recruited, who received 1 × 10^8^ deuterium-labeled Tregs. The labeled cells in circulation were reduced after 4 weeks, while skin biopsies showed a marked increase in tissue Tregs and IL-17 production by both CD4 and CD8 cells. Along with the aforementioned, to date (end of November 2018) more studies underway (Table 1). **NCT03239470** is a phase I open-label trial evaluating the infusion of a single dose (2.5 × 10^8^ vs. 10 × 10^8^ cells) of sorted autologous polyclonally expanded Tregs in adult patients with active cutaneous pemphigus. **NCT03011021** is a phase I/II randomized open-label study, led by Central South University Changsha, evaluating the infusion of 2 × 10^6^/kg of umbilical cord blood Tregs along with liraglutide therapy in adult and elder patients with autoimmune diabetes. Patients will be allocated in four groups: Tregs+liraglutide+insulin, Tregs+insulin, liraglutide+insulin, insulin alone. The same center is leading another randomized, open-label phase I/II trial **(NCT02932826)** with the aim to compare, in T1D, the infusion of umbilical cord blood Tregs combined to insulin and insulin therapy alone. **NCT02704338** is a phase I/II open-label study on the infusion of a single dose of 10-20 x 10^6^/kg autologous *ex-vivo* polyclonally expanded Tregs in patients aged 10-70 with autoimmune hepatitis (Nanjing Medical University). **NCT03185000** (TRIBUTE) is a double-blind, placebo-controlled trial (King's College London), evaluating the infusion of sorted and polyclonally expanded CD4^+^CD25^+^CD127^low^CD45RA^+^ Tregs in adult patients diagnosed with moderate to severe Crohn's Disease not tolerating or responding to at least 2 standard treatments. Patients are divided in two groups, one receiving Tregs at week 0 and placebo at week 8, the other receiving placebo at week 0 and Tregs at week 8. Doses range from 0.5–1 × 10^6^/kg, up to 8–10 × 10^6^/kg.

**Table 1:** Ongoing clinical trials adopting tregs in autoimmunity.

**Study ID**	**Phase**	**Indication**	**Enrollment/Age**	**Product**	**Dose**	**Status**
ISRCTN06128462	I	Type 1 Diabetes	12/ range 5–18	Polyclonally expanded tTregs (A)	10 and 30 × 10^6^/kg	Completed
NCT02691247	II	Type 1 Diabetes	113/ range 8–17	Polyclonally expanded tTregs (A)	2.5 and 20 × 10^6^/kg	Active, not recruiting
NCT02772679	I	Type 1 Diabetes	16/ range 18–45	Polyclonally expanded tTregs (A)	3 and 20 × 10^6^/kg	Recruiting
NCT02428309	I	Cutaneous Lupus	NA/ range 18–60	Polyclonally expanded tTregs (A)	1, 4 and 16 × 10^8^	Active, not recruiting
NCT03239470	I	Pemphigus	12/ range 18–75	Polyclonally expanded tTregs (A)	2.5 × 10^8^ and 10 × 10^8^	Recruiting
NCT03011021	I/II	Type 1 Diabetes	40/ >18	Polyclonally expanded tTregs (UCB)	2 × 10^6^/kg	Recruiting
NCT02932826	I/II	Type 1 Diabetes	40/ range 6–60	Polyclonally expanded tTregs (UCB)	2 × 10^6^/kg	Recruiting
NCT02704338	I/II	Autoimmune hepatitis	30/ range 10–70	Polyclonally expanded tTregs (A)	10–20 × 10^6^/kg	Unknown
NCT03185000	I/II	Crohn's Disease	20/ range 18–80	Polyclonally expanded naive tTregs (A)	0.5–1, 3–5 and 8–10 × 10^6^/kg	Not yet recruiting

***(A)**, autologous; **(UCB)**, umbilical cord blood*.

### Tregs in Solid Organ Transplantation

Few reports regarding the infusion of Tregs in solid organ transplantation have been published. In 2016, Todo et al. treated 10 consecutive patients with end-stage liver failure who underwent transplantation from a living donor with a cell product enriched in anergic and/or regulatory T lymphocytes (101). In detail, recipient lymphocytes and splenocytes (collected during the transplant) were cultured with irradiated donor cells in the presence of anti-CD80/CD86 antibodies for 2 weeks. Patients received a single infusion on day 13 post-transplantation. Although the cell product was contaminated by monocytes, DCs NK and B cells the numbers of Tregs infused ranged from 0.43 × 10^6^/kg to 6.37 × 10^6^/kg. From the 40 patients originally planned, the trial ended due to the acute cellular rejection during weaning in two patients with primary biliary cirrhosis and one with primary sclerosing cholangitis. Patients with no immunological related disease successfully tolerated the immunosuppression weaning started after 6 months post-transplantation and followed by a complete weaning at 18 months. Patients with acute rejection underwent on low dose of tacrolimus and mycophenolate mofetil. Although the infused cell product was contaminated with antigen specific effector cells, the authors presented this pilot study as a novel strategy for tolerance induction in patients undergoing liver transplantation for non-immunological diseases. To confirm this hypothesis, investigations are currently underway in a large group of patients excluding those ones with autoimmune disorders. In 2017, results from phase I, open-label pilot study conducted at the University of California (San Francisco) were published (102). They aim to test the feasibility of Treg isolation, expansion and infusion in kidney transplant recipients on immunosuppression with subclinical graft inflammation. Three kidney transplant recipients were enrolled according to their Kidney inflammation status detected during the 6-month post-transplant surveillance biopsy. Sorted Tregs (CD4^+^CD25^+^CD127^low^) were expanded as described above using medium containing deuterated glucose for further *in vivo* tracking. Patients received a single infusion of around 320 × 10^6^ and were maintained under tacrolimus, mycophenolate mofetil and prednisone. Follow-up biopsies were performed at 2 weeks and 6 months post-infusion. None of the enrolled patients had infusion reactions and no infections or malignancies were observed during the 1 year follow-up period. The authors showed that infused Tregs peaked in circulation the first week with deuterium signals detectable during the first month after infusion in all subjects dropping near the detection limit at 3 months after infusion. Due to the low number of the enrolled patients, it is not possible to draw any conclusion of either safety or efficacy of Treg infusion isolated from kidney transplant recipients on immunosuppression with subclinical graft inflammation. However, following the results of this pilot study new trials have been planned to test this strategy in a larger casuistic (**NCT02088931** and **NCT02711826**). In 2018, the results from the clinical trial conducted at the Northwestern University (Chicago) called TRACT have been published (93). This was a phase I dose escalation study infusing *ex vivo* expanded autologous polyclonal Tregs into living donor kidney recipients. Nine patients divided in 3 cohorts have been infused 60 days post transplantation with 0.5, 1, and 5 × 10^9^ cells, respectively. Tregs were isolated from leukapheresis collected 1 month prior to the transplant and expanded ex-*vivo* for 21 days. Patients received alemtuzumab together with the transplant for a complete lymphodepletion and 2 days before the transplant, they were placed on tacrolimus and mycophenolate. At 2 months post-transplant, prior to Treg infusion, tacrolimus was stopped and switched to sirolimus. During the follow up, no serious adverse events attributable to Treg infusion were detected and the opportunistic infection seen were linked with the immunosuppressive regimen. The authors found an increased Treg number after the infusion compared to historical control patients under the same immunosuppressive regimen. The presence of donor specific antibodies was observed in two patients but, the authors stated that this was due to the suboptimal immunosuppression. Overall, the product was safe and the authors are planning a phase II trial.

Most of the clinical trials using Tregs to prevent rejection in solid organ transplantation are still ongoing (Table 2). We are part of The ONe Study consortium where eight academic institutions along Europe and US are testing safety and feasibility of different regulatory cell populations (Tregs, tolerogenic DCs and regulatory macrophages) in kidney transplant patients. The ONe Study UK **(NCT02129881)** involved our institute (King's College London) and Oxford University; autologous Tregs have been isolated from PB, magnetically enriched, polyclonally expanded and then infused with no adverse effects in 12 patients. Following the positive experience of this trial, a phase IIb trial (The TWO study **ISRCTN11038572**) will start at the end of 2018. In this new study, 34 renal transplant recipients will be enrolled and infused with expanded Tregs 6 months after transplantation. The primary outcome will be the incidence of acute rejection episodes at 12 months post-transplantation. The ONe Study-Charité in Berlin **(NCT02371434)** is also evaluating polyclonally expanded Tregs while, the group in Milan is testing the effects of Antigen-specific Tr1 (T10 cells). Tregs specific for the donor alloantigens (DarTregs) have been tested by the US-partner of the ONe study (University of California, **NCT02244801** and Massachusetts General Hospital, **NCT02091232**). In California, sorted Tregs have been co-cultured firstly with donor B cells activated using CD40L and then re-stimulated using antiCD3/CD28 coated-beads (96). In Boston, PBMCs were co-cultured for 72hrs with an equal number of irradiated kidney donor PBMCs (first-party stimulators) in the presence of belatacept (CTLA4 blocking Ig) and then re-stimulated with new first-party stimulator without co-stimulatory blockage (103). In both trials, patients have been divided in 2 cohorts receiving 300 × 10^6^ and 900 × 10^6^ of darTregs respectively 10 days after the transplant. The Russian State Medical University in Moscow is leading a phase I clinical trials where two doses of Tregs will be infused in pediatric patients after kidney transplantation **(NCT01446484)**. Patients will be treated at day−21,−14 and the day of the transplant with alemtuzumab (monoclonal antibody specific for CD52). On day 0, patients will receive either tacrolimus or cyclosporine followed by mycophenolate mofetil at day 3. Sirolimus will start 1 month after the transplant together with the first infusion of Tregs. The second dose will be administrated after 3 months post-transplant. Although this clinical trial was supposed to end in 2014, no results are available. Other two clinical studies in the US (**NCT03284242** and **NCT02145325**) are testing safety and feasibility of Tregs after kidney transplantation. The ThRIL **(NCT02166177)** is a Phase I/IIa clinical trial conducted at King's College London. We aimed to test polyclonally expanded Tregs in liver transplant patients. The last patient was infused in 2017 and early data are already available. Two different multicentric studies running at the University of California (San Francisco), Northwestern University, (Chicago), and Mayo Clinic (Rochester) are testing DarTregs in liver transplant recipient. The first one **(NCT02474199)** aims to test safety and feasibility of DarTregs infusion only, while in the second **(NCT02188719)** they aim to infuse Tregs and reducing the use of calcineurin inhibitors. In **NCT02474199**, patients will receive a target dose of 400 × 10^6^ darTregs infused intravenously while in **NCT02188719** four cohorts of patients will receive none, 50 × 10^6^, 200 × 10^6^, 800 × 10^6^ darTregs, respectively. Both studies are still recruiting and results will not be available soon. In August 2018, at the Massachusetts General Hospital a new single-center, open-label, non-randomized clinical trial started **(NCT03577431)**. In this phase I/II study they aim to use Tregs to facilitate immunosuppression withdrawal in liver transplant recipients. Similarly to what has been used for the kidney recipients in the ONe study, a cell product containing donor-specific hyporesponsive cells in association with allospecific Tregs will be used. This cell product is generated in mixed leukocyte reaction where donor and recipient cells are co-cultured in the presence of belatacept. They have planned the infusion of 2.5 × 10^6^ up to 500 × 10^6^ cells in nine patients.

**Table 2:** Ongoing clinical trials adopting Tregs in transplantation.

**Study ID**	**Phase**	**Indication**	**Enrollment/Age**	**Product**	**Dose**	**Status**
NCT02145325	I	Living donor kidney transplant	10/ range 18–65	Polyclonally expanded tTregs (A)	0,5, 1, 5 × 10^9^	Active but not recruiting
NCT02129881	I/II	Living donor kidney transplant	12/>18	Polyclonally expanded tTregs (A)	1, 3, 6 × 10^6^/kg	Completed
NCT02371434	I/II	Living donor kidney transplant	9/ range 18–65	Polyclonally expanded tTregs (A)	0,5, 1, 3 × 10^6^/kg	Unknown
NCT02244801	I/II	Living donor kidney transplant	16/ range 18–70	Donor-alloantigen-reactive tTregs (A)	300 and 900 × 10^6^	Completed
NCT02091232	I/II	Living donor kidney transplant	8/>18	Belatacept-conditioned tTregs (A)	300 and 900 × 10^6^	Active, not recruiting
NCT02166177	I	Liver transplant	9/ range 18–70	Polyclonally expanded tTregs (A)	0.5–1 and 3–4.5 × 10^6^/kg	Completed
NCT02188719	I	Liver transplant	24/ range 21–70	Donor-alloantigen-Reactive Tregs (A)	50, 200, 800 × 10^6^	Recruiting
NCT02088931	I	Living donor kidney transplant	3/ range 18–50	Polyclonally expanded tTregs (A)	320 × 10^6^	Unknown
NCT02474199	I	CNI reduction in liver transplant	18/ range 18–70	Donor-alloantigen-Reactive Tregs (A)	400 × 10^6^	Recruiting
NCT02711826	I	Subclinical Inflammation in Kidney Transplantation	40/>18	Donor-alloantigen-Reactive Tregs (A)	1 × 10^6^/kg	Recruiting
NCT01624077	I	Liver transplant	1/ range 10–65	Induced Tregs (A)	1 × 10^6^/kg	Unknown
ISRCTN11038572	IIb	Living donor kidney transplant	136/>18	Polyclonally expanded tTregs (A)	5–10 × 10^6^/kg	Not yet recruiting
NCT01446484	I	Kidney transplant (children)	30/ range 1–18	Polyclonally expanded tTregs (A)	200 × 10^6^	Unknown
NCT03577431	I/II	Liver transplant	9/ range 17–70	Belatacept-conditioned tTregs (A)	from 2.5 to 500 × 10^6^	Not yet recruiting
NCT03284242	NA	Kidney transplant	12/ range 18–65	Polyclonally expanded tTregs (A)	NA	Not yet recruiting

***(A)**, autologous**;** NA, not available*.

## Future Directions

Although preclinical studies have shown the capacity of Tregs to treat autoimmune diseases and prevent graft rejection, in the clinic we are still far away from these ultimate goals. The first clinical studies have shown the safety and feasibility of Tregs infusion and new phase II trials are now starting or being planned. The next steps will be crucial to define a standardized strategy for treating autoimmune disease and graft rejection. One important aspect is represented by the immunosuppressive regimen used to dampen the immune response. We have recently shown that immunosuppressive drugs like tacrolimus, mycophenolate and methylprednisolone reduced Tregs' viability and proliferation in a dose dependent manner (104). Therefore, the immunosuppressive regimen adopted might have an essential role for the efficacy of the Tregs therapy. This is the reason why different strategies are now under investigation with the aim to tailor the immunosuppressive regimen to the Tregs or find the best timing for their infusion. As described in the previous section, the infusion of Tregs can be executed when patients are under rapamycin treatment. Differently to the other immunosuppressive drugs, rapamycin favors the expansion of Tregs both *in vivo* (105) and *in vitro* (90, 92) supporting their action. We believe that Tregs need to be injected in combination with other therapies tailored to the type of disease that is to be targeted. In other words, combined therapy protocols might represent a winning strategy for the future. To date, low doses of IL-2 have been used for expanding endogenous circulating Tregs in autoimmunity (106) and GvHD (107) directly *in vivo*. The main issues of this approach are represented by both the half-life of the IL-2 in circulation together with the possible activation of other detrimental cells like NK or eosinophils. New clinical trials (**NCT03556007**, **NCT03221179**, and **NCT03451422**) are testing molecularly engineered IL-2 with an increased half-life. This will allow the minimization of the dose of IL-2 administrated and the development of a more specific therapy for Tregs. Another promising strategy of combined therapy in autoimmunity is represented by the engagement of the TNF receptor 2 (TNFR2). TNF-blocking strategies are effective for the treatment of rheumatoid arthritis (22, 108) however the inflammatory effect of the TNF-alpha is mediated by the receptor 1 (TNFR1) while the TNFR2 has been shown to induce immune modulation and tissue regeneration. Tregs express higher levels of TNFR2 compared to other T cells and its expression has been linked with Treg suppressive ability in both mice (109) and human (110, 111). Due to the role of this receptor, TNFR2 antagonism has been suggested as a new promising strategy in cancer immunotherapy specifically in ovarian, lung, and cutaneous T cell lymphoma (112, 113). In addition, a defective TNF/TNFR2 interaction is critical for Treg functionality in autoimmunity. For this reason, the combined infusion of antigen specific Tregs together with TNFR2 agonists might be a winning strategy. In the last years, several reports have shown how chronic inflammation changed the microbiome composition which is essential for developing regulatory pathways involved in the maintenance of the immune homeostasis (114). To further reduce the systemic inflammation, the infusion of Tregs can be applied in combination with Treg-inducing microbial as fecal microbiome transplantation is not yet approved in clinic due to safety reasons. Recent studies have highlighted the importance of short chain fatty acids derived from bacteria as main factors mediating Treg induction. Butyrate has been implied in the up regulation of anti-inflammatory genes in DCs. Furthermore, it enhances histone acetylation of the *Foxp3* locus and the stability of FOXP3 protein (115). Finally, polysaccharide A and cell surface β-glucan/galactan from *Bifidobacterium bifidum* were able to induce Foxp3^+^IL-10^high^IFN-γ^low^ and Foxp3^−^IL-10^high^IFN-γ^high^ Tregs, respectively (116). For this reason, Treg-inducing microbial components can ameliorate the outcome of cell therapy protocol adopting Tregs.

Treg localization and migration represent the main challenges in the field. Cells delivered specifically to inflamed area will increase enormously the positive outcome of the cell therapy protocol based on Tregs. As already discussed above in this review, the use of ATRA during Tregs expansion has been shown to induce chemokine receptor specific for gut homing The discovery of the Th-like Tregs has opened another important avenue in selecting a population tailored to the type of disease. In autoimmunity, therapies targeting Th17-dependent pathways are associated with clinical benefits (117); in this scenario, Th17-like Tregs might be the ideal candidates for cell therapy protocols. Cardiac allograft vasculopathy after heart transplantation is linked to Th-1 and the use of Th1-like Tregs might be the ideal strategy. However, due to the low number of the Th-like Treg subpopulation, *ex-vivo* expansion is necessary. This might modify their phenotype especially if rapamycin or other drugs are added into the culture. Overall, new studies on these cells need to be conducted before including them as possible candidate for cell therapy. An important aspect that needs to be considered in developing Phase II/III trials will be the tracking of the infused cells. To date, only Tregs infused in patients with T1D have been monitored using deuterium labeling (84). However, this strategy is limited to the circulating cells and it is not possible so far to gain information on the localization of Tregs in the tissues. For this reason, future trials need to test new approaches for a more specific cell detection. One possibility is represented by the sodium-iodide symporter (NIS) a molecule expressed on thyroid follicular cells and essential for the uptake of plasma iodide (118). The NIS gene was firstly cloned in 1996 by Dai et al. (119) and it is considered safe, non-immunogenic and non-invasive. Although it mediates mainly the transport of iodide into the cells, NIS can translocate several other substrates detected using different system like PET or SPECT/CT. Transducing Tregs with NIS will be key for understanding their localization and whether they successfully reach the target organ or tissue. In addition, compared to the iron oxide nano-particles that persist after death of the labeled cell, NIS works only on living cells allowing at the same time the evaluation of cells viability *in vivo*. We have already developed a protocol for transducing Tregs with NIS (120). NIS expressing self-specific Tregs were radiolabelled *in vitro* with Technetium-99 m pertechnetate with no effects on cell viability, phenotype, and function. Moreover, we were able to detect these cells *in vivo* in the spleen of C57BL/6 mice 24 h after infusion by SPECT/CT. Lastly, enthusiasm is growing in the generation of antigen-specific Tregs by genetic engineering with chimeric antigen receptors (CARs). This new strategy [extensively reviewed in (121)] has been firstly developed in the tumor field and in 2017, the FDA approved the use of CAR T-cell for the treatment of acute lymphoblastic leukemia in children and advanced lymphomas in adults (122). In autoimmune diseases, CAR-T cells might be developed for targeting the pathological cells responsible of the autoimmune reaction (B cells). On the other hand, CAR can be transduced into Tregs to generate a population that is specific for a selected antigen. Due to the lack of self-protein exclusively expressed on the inflamed tissue, the generation of CAR specific Tregs in autoimmune diseases is challenging (123). Conversely, in transplantation, CAR-Tregs specific for MHC-I molecules have been generated. In the context of HLA-mismatched transplant, HLA class I specific-CAR-Tregs will target the transplanted organ without interfering with the recipient-immune system. We have shown how HLA-A2 (MHC class I protein) specific CAR Treg have the capacity to prevent skin-graft rejection in a mouse model compared with polyclonally expanded Tregs (124). However, before being tested in the clinic, CAR-Treg stability is another issue to be solved together with cell homing capacity. In fact, as CAR-Tregs are antigen specific, they do not need to migrate into the lymph node but can be specifically directed into the target organ/tissue.

## Conclusions

So far, many of the original questions for the use of Tregs in transplantation and autoimmunity remain unanswered. Results from the ongoing clinical trials will be crucial to better understand the tolerated Treg dose, timing of infusion and the immunosuppressive regimen to preserve/favor them. However, few data will be available on Tregs efficacy and whether or not we should engineer them for being antigen-specific or expressing molecules linked with the migration into the target tissue/organ. A big step forward to understand the real potential of Treg-based cell therapy will be there *in vivo* tracking. However, due to the high costs of cellular engineering and ethical approval this will not be revealed soon. For this reason, in the near future, the best strategy is represented by the combined therapy whereby antigen-specific Treg will be infused together with either low dose of IL2, rapamycin or in the future TNFR2 agonists.

## Author Contributions

MR and GF participated in manuscript writing and editing. CA and GG contributed to manuscript writing and figure development. GL contributed to manuscript editing.

### Conflict of Interest Statement

The authors declare that the research was conducted in the absence of any commercial or financial relationships that could be construed as a potential conflict of interest.
